# Multi-criteria evaluation in paradigmatic perspectives of agricultural environmental management

**DOI:** 10.1016/j.heliyon.2019.e01229

**Published:** 2019-02-15

**Authors:** Mahsa Fatemi, Kurosh Rezaei-Moghaddam

**Affiliations:** Department of Agricultural Extension and Education, School of Agriculture, Shiraz University, Shiraz, Iran

**Keywords:** Environmental science, Sociology

## Abstract

Agriculture is one of the primary activities that affects the environment due to natural resources consumption. Therefore, systematic environmental management for the agricultural sector is required. This study was conducted to analyze the paradigmatic perspective and strategies of agricultural environmental management in Iran. Considering basic criteria of environmental management, three paradigms of frontier economics, eco-development and deep ecology were compared using Analytical Hierarchy Process (AHP). The AHP, is a multi-criteria decision making techniques which is useful when there are different alternatives or indicators in decision making. Comparisons were based on the viewpoints of 117 policy makers, superior managers and main elites and agriculture sector researchers. Environmental managerial strategies also have been studied. Findings revealed paradoxes among the paradigmatic perspectives and selected strategies of different agricultural stakeholders which reduce their effective interactions. Frontier economics is the dominant viewpoint of key agricultural policy makers and other governmental executives. They prefer independent reactive strategies to cope with environmental challenges. Agricultural researchers and private sector authorities believe in eco-development. They have selected cooperative proactive strategies in this regard. Finally, deep ecology has the highest priority according to environmental specialists, who endorse strategic maneuvering and believe in modifying, rethinking and redesigning previous strategies. A paradigm shift, as well as consistency between paradigmatic perspectives and executive strategies, is suggested.

## 1 Introduction

Sustainability has been emphasized due to the imbalanced development of human societies, especially after the industrial revolution, and the negative consequences of population growth, capitation and consumption patterns (Wackernagel and Yount, 2000). As a response to the environmentally and socially destructive practices of post-war mechanization and intensification, the concept of sustainable agriculture has become prominent in research, policy, and practice (Rose et al., 2019; Janker et al., 2018). A comprehensive managerial system is needed in order to harmonize between Environmental restrictions and human needs. Co-management is kind of suitable system refers to an institutional mechanism in which government representatives and resource user-groups, such as local and indigenous communities interact to negotiate formal agreements on the distribution of rights, power, responsibilities and benefits in the resource management process (Akamani and Hall, 2019). Adaptive collaborative management (adaptive co-management) may help to improve adaptability and resilience and to develop ‘no-regret strategies’ for a sustainable management (Ehrhart and Schraml, 2018). Environmental management is a collection of managerial activities including environmental planning, environmental conservation, evaluation of the environment, legislation, monitoring and control of environmental activities (He et al., 2012; Sakr et al., 2010).

Agriculture is one of the primary activities that affects the environment due to natural resources consumption. Therefore, systematic environmental management for the agricultural sector is required. Environmental management of agriculture is defined as a balance between natural resources' capacity called biocapacity (Fatemi et al., 2018; Borucke et al., 2013) and the amount of agricultural activities. Given the limited capacity of natural resources, utilization should be rational, reasonable and calculated in order to prevent or reduce degradation of natural resources. The sustainability of natural resources depends upon our paradigm for the relationship between society and environment and the stakeholders' perspective towards the resources (Fatemi, 2017). The environmental behavior of each individual depends on how he/she thinks about natural resources. The paradigmatic viewpoint is defined as a framework for viewing the universe. It is the intellectual foundation including the values, beliefs and norms of the individual, organization or nation (Raum and Potter, 2015). It shapes the individual's attitudes and behaviors towards the environment as well as other aspects of the whole managerial system. Thus, modification of the dominant paradigm of the individual or the society would be constructive as the principal solution to improve the behaviors of that society.

Individual scientists have categorized environmental management in differing ways. De Vries (1989) proposed five paradigms which he labeled as Technocrat-Adventurer, Partners, Manager-Engineer, Steward and Cultural. Similarly, Colby (1991) classified different paradigmatic viewpoints of environmental management into five categories much more explicitly defined and much evolutionary in terms of described relationship among them that include Frontier Economics (the same as technocrat-adventurer), Environmental Protection (close to steward), Resource Management (the same as manager-engineer), Deep Ecology (identified closely with partners) and Eco-Development (mixture of cultural and steward). Recently, Aral (2005) categorized the paradigms of environmental management into Frontier Economics, Radical Environmentalism, Sustainable Environmental Management, Selective Environmentalism and Resource Allocation. These paradigmatic perspectives, contain some overlaps; for instance, environmental protection and steward are so close to frontier economics in which economic factors have been the highest priority. Resource management, manager-engineer and sustainable environmental management are similar to eco-development. It is hard to draw a specific line between these paradigms.

In the next level, the strategies of environmental management are shaped by the dominant paradigmatic perspectives of the executive managers in this area. The relationship between the organization and the external environment is emphasized to identify the strategies. So, organizations should change to the passive-reactive entities due to their exterior environment. In contrast, organizations must implement diverse strategies in order to rectify existing environmental situations, so they would become proactive entities of change by trying to manage their exterior environments. Strategies under a concept of environmental management discussed by different scientists (Leigh and Li, 2015; Lee and Rhee, 2005; Fatemi, 2017). Independent strategies are means by which the organization can reduce environmental uncertainty and dependence relying on its resources and creativity. These strategies are implemented regularly by individual organizations in an attempt to modify their competitive environments. Cooperative strategies involve implicit or explicit cooperation with other elements in the environment. In some situations, two or more organizations may implement cooperative environmental management strategies. Cooperative strategies are selected by many organizations on the assumption that combined action reduces risks and costs to individual organizations while increasing their power. Strategic maneuvering includes strategies designed to change or alter the task environment of the organization. These strategies under their related paradigms represent conscious efforts by a government or organization to change the task environment in which it operates. In fact, environmental management contains three different strategies at different influential levels: “reactive end of pipe pollution control, proactive reusing, remanufacturing, and recycling of products and materials” (Leigh and Li, 2015).

A multiple criteria evaluation of different paradigmatic perspectives of environmental management with an emphasis on the agricultural sector, as well as comparing diverse managerial strategies of the environment, are the main purposes of this study. Three paradigms of *frontier economics, deep ecology* and *eco-development* have been studied in terms of the significant differences in their main principles and assumptions. Frontier economics is the basic economic perspective; in contrast, deep ecology is the radical environmental viewpoint and eco-development is an intermediate paradigmatic perspective placed in the middle of the spectrum. Therefore, different alternative paradigms of environmental management have been introduced and then compared in terms of the perspectives of main policy makers, superior managers, elites and researchers of agricultural extension in Iran using AHP. In the next level, appropriate categories of environmental management strategies were studied and ranked based on to the viewpoints of different groups of the study.

### 1.1 Alternative paradigms

#### 1.1.1 Frontier economics

One of the basic principles of this paradigm is that natural resources and ecological services are regarded as “free gifts” of nature. The other principles include the assumptions of infinite substitutability between inputs to production, the reversibility of equilibrium and resource use, that efficient allocation through the price mechanism brings a kind of social justice by rewarding each factor according to its marginal productivity (Azam, 2016). According to this paradigm, nature is seen as a beneficial tool for humans, to be consumed, manipulated and changed for the betterment of the human life quality (Singh, 2015). In this worldview, the nature and society are seen as two separate things which humans could overcome the environment. Indeed, regarding to this viewpoint, environment should have reformed due to the human's insight and become desirable for the man's requirements. So, the human-nature relationship is seen as one-sided oriented and in a sense, zero-sum (Gendron, 2014).

Environmental management decisions are designed to use efficient technological means to realize such growth. Regarding this paradigm, many technologies could be seen as strategies for the environment management, while they were provided for human's power incensement in order to exploit production from nature, and/or to reduce the negative impacts of environment's variations on community. According to frontier economics, sustainability is not an important matter and the future is created through a price system based on free choice. There are some policy strategies based on this paradigm: “free market” means governments act only as necessary to deal with inevitable market deficiencies; “technological optimism” can be seen in this paradigm which means technology is a, progressive, and can treat any challenge it makes; and there is no need to pre-market appraisal of technology. Based on the “end-of-pipe strategy”, it is considered that nature degradation could be remade where necessary after development has proceeded to some point where clear environmental management can be afforded.

#### 1.1.2 Deep ecology

The deep ecology has been cited in the opposite side of frontier economics in spectrum of different paradigmatic viewpoints. These specialists have a whole different value system based on ethics and aesthetics rather than the financial and material orientation of economics. Deep ecology fundamentally rejects the dualistic view of humans and nature as separate and different (Pepper, 2003). This paradigm involves a radical philosophy which attempts to redefine the relationship between humans and nature by granting ethical status to animals, plants, ecosystems, and even the non-living parts of our natural environment. It values nature (non-human and even non-living elements) for its own sake and judges that nature deserves protection because of its intrinsic value (Pentreath, 2004).

The beliefs of deep ecology proponents are in opposite of anthropocentrism*.* The basic deep ecology tenets concerning this fundamental relationship are intrinsic biospecies equality, major reductions in human population, bioregional autonomy, promotion of biological and cultural diversity, decentralized planning utilizing multiple value systems, and non-growth oriented economics (Colby, 1991; Fatemi et al., 2018). Additional tenets are the dependence on simple technology, focusing on the use of indigenous management and technological systems. Deep ecologists see technological fixes as usually leading to larger, costlier and more difficult problems (Paterson, 2006). Sustainability is the wrong question due to this paradigmatic viewpoint as it turns out of man centeredness. There are some policy strategies in this paradigm including shifting the man from egocentric view toward a harmony with nature as well as technology management which means accepting only those clean technologies with no harmful effects on the environment. Deep ecologists are highly resistant to the idea of using economic costing measures for environmental damage. They believe that ecological values exist independent of values held by society, and imply an infinite value for living resources and even some non-living ones.

#### 1.1.3 Eco-development

The concepts of eco-development began to emerge as an alternative in an attempt to explicitly incorporate cultural, social and ecological goals into development (Sachs, 2000). Three eco-development objectives are social equity, ecological sustainability, and economic viability. Considering the principles of self-sufficiency and participation were subsequently added as criteria for eco-development besides the satisfaction of basic needs (Glaeser, 2000). The basic premise of the eco-development paradigm is that all aspects of the political, economic, sociocultural, and techno-scientific subsystems need to be ecologized, rather than just the economic and technological subsystems. Industrial ecology, agro-ecology, broader community participation in development planning, utilizing more local knowledge, more committed, cooperative relations between the public and private sectors, sociotechnical systems design criteria, new types of land use regimes such as extractive forest reserves and game ranching, and synergetic integrations of agriculture, industrial, and energy systems are some of the means of this paradigm (Colby, 1991).

This paradigm argued an alternative notion of development as a policy including three main components: needs, self-reliance and environment. It was declared that development and the nature form a “dialectical union” (Glaeser, 2000). The core of the eco-development paradigm is to reform the connection between society and environment into a “positive sum game” by rearranging man activities to be synergetic with ecosystem processes (Colby, 1991). The use of “development” implies a clear reorientation and advancement of the level of integration of social, environmental and economic concerns. Eco-development underlines longer term management of “adaptability”, “resilience”, and “uncertainty” to reduce the ecological stresses (Konchak and Pascual, 2006). This paradigm moves on from economizing ecology to ecologizing the economy or whole social systems. Ecologize economy, moral change to gradually generate environmental concerns, technological realism, precautionary principle to manage uncertainty, life cycle framework, product policy, pollution pays and policy equity are some policy strategies in relevance with the eco-development paradigm (Rezaei-Moghaddam et al., 2005). One goal of eco-development is to eliminate the need for the polluter to pay by reforming the economy according to ecological principles to regularly pollution reduction, rather than just to fit pollution control economically and efficiently into existing structures.

## 2 Methodology

### 2.1 What is AHP?

The AHP, proposed by Thomas L. Saaty, is one of the most applied multi-criteria decision making techniques (Saaty and Vargas, 2006). It is useful when there are different alternatives or indicators in decision making. Indicators could be quantitative or qualitative. AHP has been used for analyzing unstructured challenges in a variety of decision making conditions ranging from simple personal decisions to complex, capital intensive decisions in numerous areas containing management, economics and politics. The AHP technique is based on pair-wise comparisons.

Generally, the ranking and prioritization of the alternatives in AHP includes four phases (Saaty and Vargas, 2006): (1) Making a hierarchical model, in which all of the elements and sub-elements involved in the decision making process should be placed between the main goal and the alternatives in different levels; (2) Making pair-wise comparisons in which each element is compared pairwise with the other elements and scored based on the comparison; (3) Weights calculation in which the primary weight is assessed based on the comparison of each element's score with the others' named relative weight. In this respect, there are four different methods including row sum, column sum, geometric mean and arithmetic mean (Ghodsi-Pour, 2002; Chen and Huang, 2004). Final weight is assessed by the aggregation of all the weights; and (4) Consistency of the system in which the inconsistency rate is the mechanism determining the validity of the responses from pairwise comparisons. Since all of the AHP calculations are based on primary judgments of the respondents in a pairwise comparison matrix, the existence of any errors and inconsistency in prioritizations will be defaced in the final output. The entire calculation process of AHP can be performed by different software programs such as Expert Choice or Super Decisions.

### 2.2 Research steps

The entire research process from conception to design of the research instruments is described step by step as follows.

#### 2.2.1 First step

Different components of environmental management have been extracted from the literature based on diverse paradigmatic viewpoints. In this phase, a total of 63 components was listed as indicators of environmental management. Then the similar ones were merged and new appropriate concepts were replaced based on the research team. Finally, 35 components in 4 main groups were categorized after incorporating and summarizing the first list (Table 1).

**Table 1:** Results of ranking of agricultural environmental management components.

Category	component	Reference	Mean	Rank
Economic	Economic dependency on natural resources	Rientjes (2002); Redclift (2006); Kapoor (2001).	4.53	1
	Human basic needs	Adams (2009); Rientjes (2002); Virapongse et al. (2016).	4.22	2
	Environmental taxes (Green taxes)	Singh (2015); Colby (1991); Grossman (2007).	4.05	3
	Employment	He et al. (2012); Redclift (2006).	3.76	4
	Economic growth	Virapongse et al. (2016); Kapoor (2001).	3.05	5
	Self-sufficiency	He et al. (2012); Rientjes (2002).	2.76	6
	Private possession of resources	Rose et al. (2019); Colby (1991); Walker et al. (2006).	2.41	7
	Combination of resources possession systems	Grossman (2007); Janker et al. (2018); Colby (1991).	2.29	8
	Risk management	Lee and Rhee (2005); Grossman (2007).	2.05	9
Social-Cultural	Improvement of environmental culture and awareness	Sakr et al. (2010); Pentreath (2004).	4.82	1
	Equity and poverty alleviation	Rezaei-Moghaddam et al. (2006); Kapoor (2001).	4.41	2
	Mutual collaboration and participation	Colby (1991); Ragkos et al. (2015); Sakr et al. (2010); Berkes (2009).	4.29	3
	Social institutionalization and environmental organizations	Sakr et al. (2010); Berkes (2009); Janker et al., (2018), Ragkos et al. (2015).	4.17	4
	Environmental attitudes	Adams (2009); Rezaei-Moghaddam and Fatemi (2013); Ragkos et al. (2015).	3.94	5
	Life quality of stakeholders	Buttel and Humphery (2002); Rezaei-Moghaddam et al. (2005).	3.65	6
	Social equity	Kapoor (2001); Virapongse et al. (2016); Buttel and Humphery (2002).	3.58	7
	Indigenous knowledge and experiences	Berkes (2009); Mavhura et al. (2013).	3.41	8
Environmental	Biodiversity	Paterson (2006); Pentreath (2004); Wang et al. (2012).	4.47	1
	Rational use of natural resources	Passeri et al. (2013); Moore et al. (2012); Hoekstra (2009); Kitzes and Wackernagel (2009); Wiedmann and Barrett (2010).	4.23	2
	Prevention of resources degradation	Kissinger and Gottlieb (2012); Fatemi et al., 2018.	4.11	3
	Reduction of environmental pollutions	Adams (2009); Paterson (2006).	4.05	4
	Development of clean energies extraction	Walker et al. (2006); He et al. (2012).	3.94	5
	Biocapacity and natural resources thresholds	Borucke et al. (2013); Galli et al. (2014); Wackernagel (2005); Wang et al. (2012); Galli et al. (2012).	3.82	6
	Renewable resources management	Nemat Pour and Rezaei-Moghaddam (2014).	3.53	7
	Ecologic resilience	Malek-Saeidi and Karami (2013); Walker et al. (2006); Malek-Saeidi et al. (2016); Berkes et al. (2003).	3.17	8
	Non-renewable resources recycling (Waste management)	Glaeser (2000); Colby (1991).	2.82	9
Technological-Political	Environmental adaptability	Colby (1991); Virapongse et al. (2016).	4.87	1
	Eco-friendly technologies	Salehi et al. (2008); Durant et al. (2004).	4.83	2
	Biotechnology in agriculture	Salehi et al. (2012); Walker et al. (2006).	3.70	3
	Non-use of chemical inputs in agriculture	Durant et al. (2004); Leigh and Li (2015).	3.29	4
	Integrated pest management	He et al. (2012); Glaeser (2000); Durant et al. (2004).	3.17	5
	Minimum-use of inputs in farming	Mercati (2016); Colby (1991); Salehi et al. (2012).	3.06	6
	Modern agricultural technologies for yield increase	Mercati (2016); Rezaei-Moghaddam et al. (2005).	2.94	7
	Decentralization in implementation (Localization)	Leigh and Li (2015); Lin et al. (2016); Akamani and Hall. (2019); Adam and Eltayeb (2016).	2.76	8
	Returning to the traditional agriculture	Anaya and Huber-Sannwald (2015).	1.82	9

Scale of the components: (1–5).

#### 2.2.2 Second step

The preliminary questionnaire including 35 components extracted from the literature in the first step (components of Table 1) was designed. Trying to gather the viewpoints of different groups of experts, scientists and scholars of environmental management in agriculture, a total 62 questionnaires were completed by different groups including 10 managers and specialists of the Central Office of Environmental Protection of Fars province and 20 elites of the think tank of this Office, 12 professors of the School of Agriculture of Shiraz University (Departments of Environment and Natural Resources and Agricultural Extension and Education) as well as the Global Footprint Network in the U.S.A., 10 members of the Council of Engineering System Organization of Agriculture and Natural Resources of Fars province and 10 managers and executives of the Organization of Agriculture Jihad of Fars province.

The respondents were asked to rank and weight the components of each category in the spectrum of 1–5 based on the importance of each component in sustainable environmental management of agriculture. Based on Q-methodology (Doody et al., 2009; Forouzani et al., 2013) they also were requested not to assign the same weight to the majority of the components, so that the same weight could not be assigned to more than two components of every category. The scientists were asked to merge similar components or add new ones which did not exist in the questionnaire. The weighting results of the elements of environmental management in agriculture with the rank of each component is shown in Table 1.

#### 2.2.3 Third step

According to the ranking results of the previous step, the 9 ultimate criteria of environmental management in agriculture were selected in order to design the main research questionnaire using AHP (Table 2). These criteria were designed in a special questionnaire in the format of a pairwise matrix to compare two by two regarding the priority of each criterion in the spectrum of 1–9. The total 117 questionnaires were completed by 5 different groups of policy makers, decision makers and managers of environment and agricultural sector who have the key role in environmental and agricultural policy making of the country. These five groups are described below.

**Table 2:** Conceptual definitions of final criteria of agricultural environmental management.

No.	Criteria	Conceptual definitions
1	Human basic needs	Trying to meet the basic needs of the farmers with emphasis on economic needs in order to improve the life quality and livelihood of the rural people.
2	Economic dependency on natural resources	Dependency of the farmers and rural society on natural resources utilization just for income.
3	Environmental ethics and culture	Emphasis and concentration on ethical and spiritual aspects and improvement of environmental awareness as well as better resource conservation by appropriate education and effective advertising in rural areas and other agricultural stakeholders.
4	Rational use of resources	Wise and rational use of resources regarding biocapacity and natural resources thresholds to ensure that the farmers' consumption does not exceed the regeneration capacity of the resources.
5	Equity and poverty alleviation	Justice and balance in distribution of the facilities and natural resources availability among all of the farmers as well as socio-economic poverty alleviation.
6	Eco-friendly technologies	Utilization of technologies, tools, agro-instruments, inputs and methods which not only improve the quantity and quality of the products, but also do not damage the environmental and natural resources capability.
7	Biodiversity	Conservation of different kinds of plant species and diverse varieties with various needs in order to adapt to different climatic conditions including probable conditions such as drought.
8	Environmental adaptability	Adaptability is defined as the response to ongoing environmental changes. It is opposite of vulnerability and leads to socio-economic resilience.
9	Mutual collaboration and participation	Maximum use of all kinds of human capacities and different stakeholders in agricultural organizations including managers, experts, extension agents, farmers and other rural people in planning, decision making and implementation of environmental activities and resource conservation.

##### 2.2.3.1 National key policy makers of Agricultural Extension at the Ministry of Agriculture Jihad (AEMAJ)

The Ministry of Agriculture Jihad as the main trustee of acting and implementing of national agricultural policies includes 7 vice chancellors of which the Extension, Education and Research deputy is one of the main ones. This group of study includes top managers and executives who are at the vice chancellor office of the Ministry of Agriculture Jihad of Iran. Indeed, they are the main policy makers and decision makers of the agricultural extension sector and have key roles in macro policies and program planning of agricultural extension activities of the country. Especially in Iran, which has the centralized government and hierarchical system, the vice chancellor of agricultural extension in the Ministry of Agriculture Jihad is in the highest place of the extension administrative chart for the whole decision making and intervention sectors of agricultural extension in Iran. Since their decisions and activities are affected by their paradigmatic viewpoints in this area, the perception of their worldview will be necessary in order to achieve sustainable environmental management in agriculture. Ten of the managers and executives of agricultural extension of the vice chancellor office of the Ministry of Agriculture Jihad of Iran were selected and their viewpoints in terms of different paradigms and strategies of environmental management were compiled and used at macro policies of the country were asked to complete a special questionnaire.

##### 2.2.3.2 Top managers of “Organizations of Agriculture Jihad (OAJ)” at the provincial level

The organizations of Agriculture Jihad of different provinces are placed after the Ministry of Agriculture Jihad in the hierarchical executive agricultural sector of Iran. These organizations control the agricultural activities at county, district and rural levels of each province, they act as a liaison between the Ministry of Agriculture Jihad and other executives sector at provincial level. The Organization of Agriculture Jihad of every province is located at the center of that province. This management system connects the agricultural executive sectors of counties and the rural areas. As for the more accurate analysis, the “Organizations of Agriculture Jihad” and their “Extension Coordination Managements” of 4 provinces of Khuzestan, Kermanshah, Bushehr and Kohgiluyeh and Boyer-Ahmad were selected in this group. Thirty-five of the presidents, managers and executives of these 4 provinces were chosen for completing the questionnaire.

##### 2.2.3.3 Top managers of “Agricultural and Natural Resources Research and Education Centers (ANRREC)” at the provincial level

Besides the Organizations of Agriculture Jihad of provinces, there are also Agricultural and Natural Resources Research and Education Centers in each province which have the responsibility of conducting agricultural research projects. For more accurate analysis, top managers in Agricultural and Natural Resources Research and Education Centers of 4 provinces of Fars, Kermanshah, Bushehr and Kohgiluyeh and Boyer-Ahmad were selected in this group. A total 32 of managers, directors and executives of these 4 provinces were selected for the questionnaires' completion.

##### 2.2.3.4 Top managers and specialists of Engineering System Organization of Agriculture and Natural Resources (ESOANR) of Fars province (private sector)

The Engineering System Organization of Agriculture and Natural Resources has been established in order to facilitate gradual privatization of agriculture in 2001 at national and provincial levels. Some of the main goals of ESOANR are developing agricultural modern knowledge and technologies, quantitative and qualitative grow of agricultural products, environmental and renewable natural resources conservation and sustainability in order to achieve sustainable development, rights protection or agricultural engineering and the integrity, coordination and collaboration among the farmers and other government and non-government agricultural sectors. Ten members of the Engineering Council of Agriculture and Natural Resources System of Fars province were selected as representatives of agricultural specialists as well as the agricultural private sector for this research.

##### 2.2.3.5 Top managers and think tank elites of the Central Office of Environmental Protection (COEP) of Fars province

The Organization of Environmental Protection is a government institution dependent on the presidential palace with the main aims of natural ecosystems conservation and the restoration of negative environmental consequences, prevention and control of environmental degradation and pollution, ecological capability evaluation in order to achieve wise and rational use of environmental resources and continuous control supervision of natural resources consumption. The offices of Environmental Protection under control of the National Organization of Environmental Protection are located in the centers of the provinces. Also in 2012, a professional group called “environmental think tank” was created in the Central Office of Environmental Protection of Fars province by the Organization of Environmental Protection of the country for the first time including 40 scientists, specialists and advanced scholars in different areas of technical, legal, educational, social, economic and policy making for determining and resolving environmental problems. The solutions produced by the environmental think tank are the constructive guide for the provincial Office of Environmental Protection. A total 30 persons including the top managers and executives (the head and deputies of the Central Office of Environmental Protection of Fars province) as well as 26 specialists of the think tank of this office comprised the fifth group of the study.

#### 2.2.4 Fourth step

Following the study and analysis of paradigmatic viewpoints of different studied groups towards environmental management in agriculture using AHP, the perspectives of agricultural policy makers, scientists, executive authorities and researchers of the same five groups were asked about appropriate strategies for effective agricultural environmental management in this step. A specific questionnaire was designed including a list of questions regarding different types of environmental management strategies (independent strategies [14 items], cooperative strategies [10 items] and strategic maneuvering [7 items]) in a Likert scale and required data was collected from the respondents. The reliability of the questionnaire was verified by a pilot study with 30 experts out of the main sample of the study from the executive experts of Extension and Service Centers of Agriculture Jihad of Kazeroon and Firouz Abad counties of Fars province. Cronbach's alphas of independent strategies, cooperative strategies and strategic maneuvering were calculated 0.78, 0.79 and 0.82, respectively. It was possible to rank different kinds of strategies due to the perspectives of different groups as well as comparing the opinions of different groups regarding the type of selected strategies.

## 3 Results and discussion

Initially, the results of paradigmatic viewpoints of five groups in terms of agricultural environmental management were presented and analyzed. Then, different types of appropriate strategies of environmental management were studied, compared and analyzed based on the perspectives of different active groups in the agricultural sector.

### 3.1 Analyzing the paradigmatic viewpoints of agricultural environmental management

**Fig. 1:**
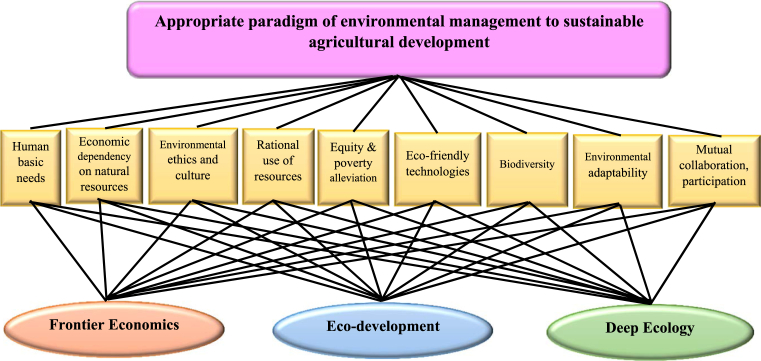
Hierarchical model for selection of appropriate paradigm of environmental management for sustainable agricultural development.

Making a hierarchical network is he first step of the AHP to present the challenge which the overall goal cited on the top, then the middle shows the criteria, and finally the bottom indicates the alternatives. In this study, the overall goal was to assess which environmental management paradigm (Frontier economics, Eco-development, Deep ecology) would be appropriate for the sustainable agricultural development of Iran. The overall objective of “selection of an environmental management paradigm for sustainable agricultural development” was placed at the top level of the analytic hierarchy shown in Fig. 1. Then, the key evaluation criteria for assessing the objective were identified. The nine described key criteria were identified previously and include human basic needs, economic dependency on natural resources, environmental ethics and culture, rational use of resources, equity and poverty alleviation, eco-friendly technologies, biodiversity, environmental adaptability and mutual collaboration and participation. Finally, three alternatives, frontier economics, eco-development and deep ecology were placed at the bottom of the AHP hierarchical model.

First, a pairwise comparison of criteria was accomplished and the following five groups were involved in the pairwise comparison of the nine criteria. The use of the AHP model requires determining the relative importance of each of the criteria in the hierarchy. Each criterion in a level is compared pairwise with other criteria at the same level, with respect to the criterion at a higher level. One hundred and seventeen participants in five groups examined the criteria with respect to the overall goal (selection of an appropriate paradigm of environmental management for sustainable agricultural development). Before the performance of pairwise comparisons, all members of the groups were given instructions on how to conduct comparisons among criteria with respect to the overall goal. Their judgment of the importance of one criterion over another can be assessed subjectively and converted to a numerical value using a scale of 1–9. Table 3 shows the normalized weights and the rank for the nine criteria with the overall goal in each of the five groups.

**Table 3:** Prioritizing and ranking of the criteria for sustainable agricultural development.

Components	AEMAJ	OAJ	ANRREC	ESOANR	COEP
Human basic needs	0.360 (1)	0.460 (1)	0.025 (8)	0.055 (6)	0.022 (8)
Economic dependency on natural resources	0.274 (2)	0.131 (2)	0.015 (9)	0.019 (9)	0.017 (9)
Environmental ethics and culture	0.039 (6)	0.032 (7)	0.049 (6)	0.025 (8)	0.253 (2)
Rational use of resources	0.105 (3)	0.072 (5)	0.048 (7)	0.217 (1)	0.063 (4)
Equity and poverty alleviation	0.090 (4)	0.115 (3)	0.117 (5)	0.199 (2)	0.044 (7)
Eco-friendly technologies	0.029 (8)	0.023 (8)	0.163 (4)	0.053 (7)	0.240 (3)
Biodiversity	0.028 (9)	0.021 (9)	0.169 (3)	0.101 (5)	0.268 (1)
Environmental adaptability	0.048 (5)	0.054 (6)	0.203 (2)	0.175 (3)	0.046 (6)
Mutual collaboration and participation	0.030 (7)	0.091 (4)	0.211 (1)	0.157 (4)	0.047 (5)
Inconsistency Ratio	0.09	0.08	0.07	0.08	0.09

Scale: if 1 = equally important, if 3 = moderately more important, if 5 = strongly more important, if 7 = very strongly more important, if 9 = overwhelmingly more important; 2, 4, 6 and 8 are intermediate values that can be used to represent shades of judgment between the five basic assessments (The ranks of each criterion are presented in parentheses).

After pairwise comparisons for all the criteria, the next step was comparisons of the sustainable agricultural development paradigms with respect to the criteria. Pairwise comparisons on the alternative paradigms (i.e., frontier economics, eco-development and deep ecology) were performed with respect to each criterion. Results verification of the decision is shown using sensitivity analysis. Thus, a sensitivity analysis was performed to determine how sensitive the alternatives are to change in the importance of the criteria. Dynamic, gradient, performance and two dimensional analyses are the five graphical sensitivity analysis modes as outcomes of AHP providing by Expert Choice.11. Performance sensitivity analysis was used in this study. It demonstrates how well each alternative performs on each criterion by changing the importance of the criterion. The results of pairwise comparisons of criteria as well as synthetizing judgments by the five groups are presented below.

#### 3.1.1 Key policy makers of Agricultural Extension of the Ministry of Agriculture Jihad (AEMAJ)

Human basic needs and economic dependency on natural resources with the weights of 0.360 and 0.274 were the two main criteria with the highest priorities based on the opinions of key policy makers working at the agricultural extension deputy of Ministry of Agriculture Jihad of Iran (Table 3). These two criteria, according to their names, focus on economic aspects and are consistent with the frontier economics paradigm. Rational use of resources (0.105) and equity and poverty alleviation (0.090) were perceived by this group as the third and fourth rank (Table 3) and the weights of environmental adaptability and environmental ethics and culture were 0.048 and 0.039, respectively (Table 3). Finally, mutual collaboration and participation (0.030), eco-friendly technologies (0.029) and biodiversity (0.028) were considered to be least important by this group and were placed in the last priorities (Table 3). The inconsistency ratio for this group's pairwise comparisons was 0.09 which is less than the tolerable level of 0.1 and acceptable (Table 3).

Based on findings, the environmental criteria of the deep ecology paradigm such as eco-friendly technologies and biodiversity were considered to be the least important by agricultural extension key policy makers of Iran. It is reasonable to see this type of weak perspective toward environmental protection by the group which constitutes the main macro policy making and decision making of agricultural extension, suggesting negative environmental consequences for agriculture of Iran. The intensification trend of environmental crisis and extended natural resources degradation would be expected due to the dominant paradigmatic viewpoints of the key agricultural extension policy makers of the country.

**Fig. 2:**
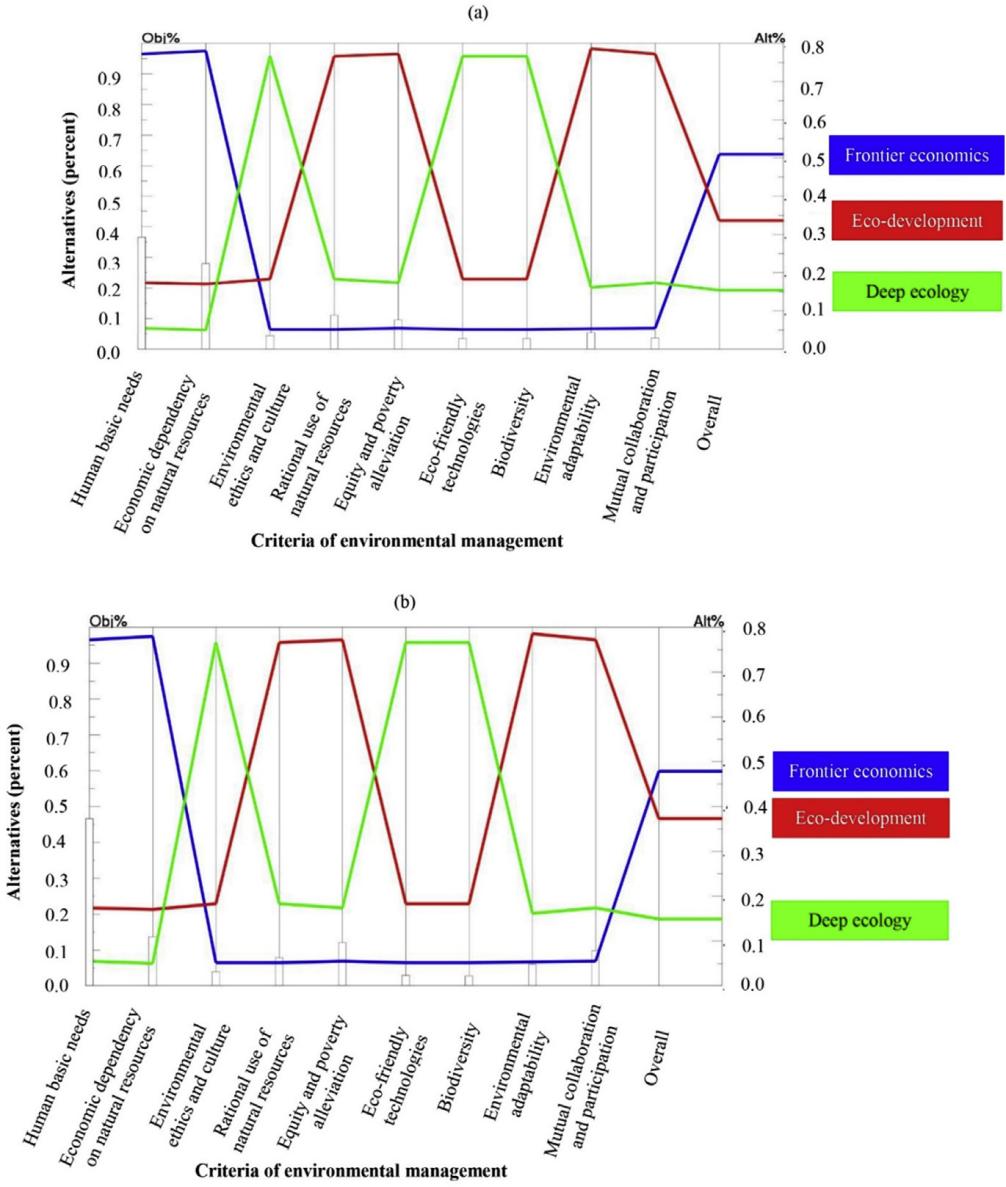
Priority of different paradigms of environmental management for sustainable agriculture as perceived by AEMAJ (a), OAJ (b).

Fig. 2(a) shows how each alternative was prioritized relative to another alternative with respect to each criterion as well as overall. Based on the findings, the key national policy makers of agricultural extension gave first rank to the frontier economics paradigmatic perspective in order to sustainable agricultural development. Eco-development and deep ecology have been placed after frontier economics. The final weights of these paradigms calculated as 0.510, 0.336 and 0.154, respectively (Table 4). According to the final weights of each paradigm, frontier economics had the highest priority by the national policy makers, eco-development had moderate importance and the environmental perspective of deep ecology had the least priority with considerable difference from the other paradigms. Indeed, economic growth and implementing the strategies to maximize agricultural production are seen as the master key to all of the agricultural challenges of Iran due to the dominant paradigmatic perspectives of national agricultural extension policy makers.

**Table 4:** Summary of results for AHP analysis of effects of environmental management models.

Alternatives	AEMAJ	OAJ	ANRREC	ESOANR	COEP
Frontier Economics	0.510 (1)	0.479 (1)	0.081 (3)	0.106 (3)	0.080 (3)
Eco-development	0.336 (2)	0.373 (2)	0.524 (1)	0.623 (1)	0.300 (2)
Deep Ecology	0.154 (3)	0.149 (3)	0.394 (2)	0.272 (2)	0.620 (1)

The ranks of each alternative are presented in parentheses.

#### 3.1.2 Top managers and authorities of Organizations of Agricultural Jihad (OAJ) at the provincial level

**Fig. 3:**
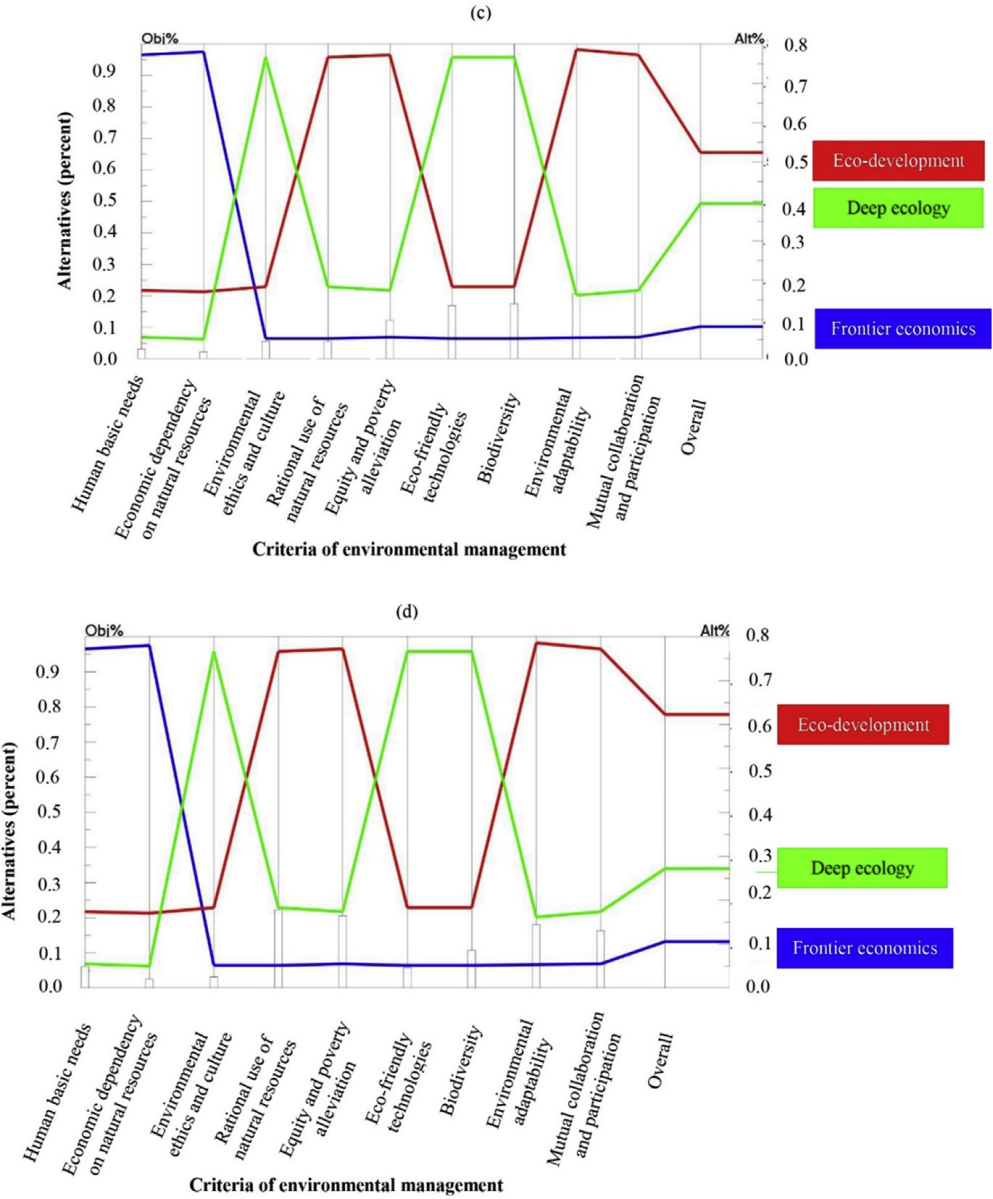
Priority of different paradigms of environmental management for sustainable agriculture as perceived by ANRREC (c), ESOANR (d).

Human basic needs with the weight of 0.460 was placed in the first rank with a high difference from the other criteria by the second group of the study (Table 3). Following that, economic dependency on natural resources (0.131) and equity and poverty alleviation (0.115) were assigned as the second and third ranks with high priority. Mutual collaboration and participation, rational use of resources and environmental adaptability had moderate importance in the beliefs of the OAJ group with the weights of 0.091, 0.072 and 0.054, respectively (Table 3). Finally, environmental ethics and culture (0.032), eco-friendly technologies (0.023) and biodiversity (0.021) were considered to have the least priority and were placed at the last ranks by the top managers and high executives of Organizations of Agriculture Jihad of four provinces of this study. As shown in Fig. 3, the inconsistency ratio of the pairwise comparisons matrix of this group was 0.08 which is acceptable statistically.

Comparison of the two groups of key agricultural extension policy makers and top managers of the Organizations of Agricultural Jihad, revealed economic criteria such as human basic needs and economic dependency on natural resources to be the main criteria for agricultural development by the both groups, as these two economic criteria were placed in the first and second ranks by the AEMAJ and OAJ groups. On the other hand, the very least priority of environmental criteria such as eco-friendly technologies and biodiversity was the second noticeable points of comparisons of these two groups. Thus, the current environmental crisis, as well as extended degradations, are not unexpected based on the purely economic perspective of key executives of the agriculture sector, especially the agricultural extension in Iran.

Top managers and executives of Organizations of Agriculture Jihad selected the frontier economics paradigmatic viewpoint as the main goal to sustainable agricultural development resembling the key agricultural extension policy makers (Fig. 2b). The total weight of this paradigm was assessed at 0.479. Eco-development and deep ecology placed in the next priorities with weights of 0.373 and 0.149 (Table 4). There is similarity between the first two executive groups which placed priority on the frontier economics paradigm and considered deep ecology as the least important paradigm with noticeable difference. Therefore, economic growth using modern technologies to increase production was considered to be the most important factor by the main managers of Organization of Agriculture Jihad of Iran following their superiors at the ministry level. In other words, the similar perspectives of AEMAJ and OAJ in ranking of the EM paradigms might be attributed to the hierarchical centralized system of government organizations of Iran. It is probable that most of the experts of these two organizations believe in environmental conservation in their personal perspective but when they put in their organizational status, they should act harmonic with the main goal of the institution and try to make decisions and policies in relevant with higher production and economic growth. Thus, the frontier economics was placed as first rank in Fig. 2, then eco-development and deep ecology were placed after with significant differences.

#### 3.1.3 Top managers and researchers of Agricultural and Natural Resources Research and Education Centers (ANRREC) of the provinces

The criteria of mutual collaboration and participation and environmental adaptability were the most important criteria perceived by this group with weights of 0.211 and 0.203, respectively (Table 3), followed by biodiversity (0.169), eco-friendly technologies (0.163) and equity and poverty alleviation (0.117) with noteworthy weights and were placed in the third to fifth ranks. The managers and researchers of ANRREC of the four provinces of this study gave moderate priority to environmental ethics and culture as well as rational use of resources. The final weights of these two criteria as shown in Table 3 were 0.049 and 0.048, respectively. The criteria of human basic needs (0.025) and economic dependency on natural resources (0.015) had the least importance in comparison with the other criteria in order to achieve sustainable agricultural development. The inconsistency ratio of pairwise comparisons of this group equals 0.07 which was less than the tolerable level of 0.1 (Table 3).

The results revealed the different opinions of the members of agricultural and natural resources research and education centers in the four provinces of the study with the two executive groups mentioned previously. This group, contrary to the other two last groups, accorded the lowest priority to the economic criteria of human basic needs and economic dependency on natural resources, but considered the socio-environmental criteria such as mutual collaboration and participation, biodiversity and eco-friendly technologies as highly important.

Based on Fig. 3(c), the eco-development paradigmatic viewpoint was considered the highest priority in order to achieve the main goal of sustainable agricultural development; this was followed by deep ecology. The final weights of these two paradigms were 0.524 and 0.394, respectively (Table 4). The managers and researchers of ANRREC of the four provinces of the study selected the frontier economics with the noticeable difference and the weight of 0.081 as the least priority. Providing comprehensive environmental management to sustainable agricultural development entails the simultaneous notice to both economic and environmental aspects, as the “eco” signifies both economic and ecological factors.

#### 3.1.4 Council of Engineering System Organization of Agriculture and Natural Resources (ESOANR) of Fars province

Rational use of resources was the most important criterion in the experts' of the council of ESOANR of Fars province viewpoint. This criterion with the weight of 0.217 had the first rank among the others (Table 3). It was followed by the criteria of equity and poverty alleviation, environmental adaptability, mutual collaboration and participation and biodiversity, all of which had significant importance. The weights of these criteria were 0.199, 0.175, 0.157 and 0.101, respectively (Table 3). The priority of the two criteria of human basic needs (0.055) and eco-friendly technologies (0.053) were at a moderate level in the opinion of the experts of this group (Table 3). Finally, the criteria of environmental ethics and culture as well as economic dependency on natural resources had the least priority among the all criteria with weights of 0.025 and 0.019 (Table 3). The inconsistency ratio of the pairwise comparisons matrix of this group was 0.08 which was less than tolerable level of 0.1 and acceptable (Table 3).

The main decision makers of ESOANR of Fars province selected the eco-development paradigmatic viewpoint as an appropriate perspective toward sustainable agricultural development (Fig. 3d). The final weight of this paradigm was calculated 0.623 (Table 4). This was followed by deep ecology with the weight of 0.272 in the second rank and the frontier economics paradigm as the least important paradigmatic viewpoint perceived by the main private sector of agriculture of Iran (Table 4). Eco-development was placed in the middle of the spectrum which had two radical paradigmatic perspectives of frontier economics and deep ecology. It suggests the need to conduct future studies by an interdisciplinary research team including scientists from economic, social and environmental sciences. Resembling the environmental challenges would be possible only by comprehensive considering to all of the aspects due to the proponents of eco-development paradigm.

There were some similarities in comparing the findings of the groups of ANRREC and ESOANR of Fars province in terms of the selection of an appropriate paradigm for sustainable agricultural development. Despite differences in the final weights, the order of the paradigms was the same in both groups. Eco-development was designated the first rank in the opinion of the two groups followed by deep ecology and frontier economics, respectively. The close connection of both groups with academia, pure research and updated results of new studies worldwide, suggests that they have increased environmental awareness as well as a more rational perspective about environmental protection. Interdisciplinary tendency of the researchers in these two organizations reflects in their preferences regarding paradigmatic perspectives of environmental management. As it is shown in Fig. 3, the multi-dimensional and integrative worldview of eco-development was chosen as the first rank in the diagram. They also selected deep ecology as the second rank following the worldwide mainstream of environmental conservation and sustainability. Finally, the frontier economics was placed at the lowest part of the diagram. Following interdisciplinary tendency of the researchers in these two organizations However, a questionable point relates to weak communications between governmental executive sectors of agriculture (AEMAJ and OAJ) and the research and private sectors of agriculture (ANRREC and COEP of Fars province) in Iran. So the research subjects are come from the international literature with no relevance to the main problems of the Iran and the research findings would not be applicable for the agricultural policies and programs as well.

#### 3.1.5 Top managers and the think tank members of Central Office of Environmental Protection (COEP) of Fars province

The criteria of biodiversity, environmental ethics and culture and eco-friendly technologies were considered as the highest priorities for sustainable agricultural development based on the viewpoints of top managers and think tank members of COEP of Fars province. The weights of these three criteria were 0.268, 0.253 and 0.240 with significant difference from other criteria (Table 3). Rational use of resources placed at the fourth rank with the weight of 0.063. According to the opinion of the experts of this group, other criteria such as mutual collaboration and participation (0.047), environmental adaptability (0.046) and equity and poverty alleviation (0.044) had moderate importance. While the two criteria of human basic needs and economic dependency on natural resources with the final weights of 0.022 and 0.017 were considered to be the lowest priorities with assignment as the two last ranks (Table 3). The inconsistency ratio of the pairwise comparisons of this group was 0.09 which is acceptable (Table 3).

Based on the findings, the managers and specialists of the COEP of Fars province had an environmental perspective by prioritizing such criteria as biodiversity, environmental ethics and culture as well as eco-friendly technologies with sizeable differences from other factors. This means that they had a strong tendency toward environmental conservation as the highest priority. Indeed, they believe in special consideration for environmental criteria as the only way to attain sustainable agricultural development.

**Fig. 4:**
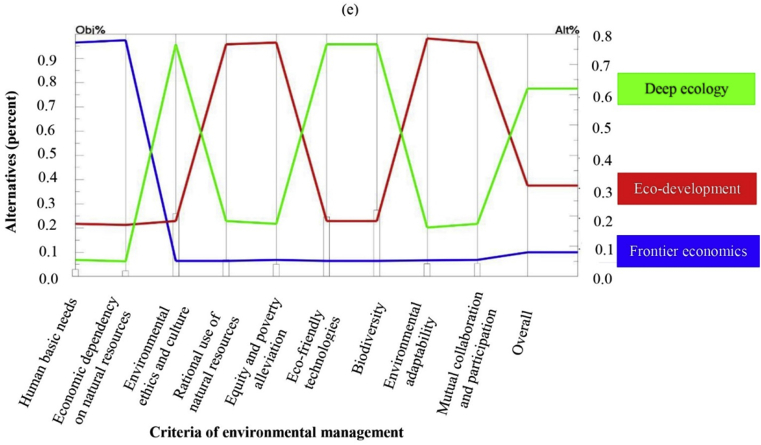
Priority of different paradigms of environmental management for sustainable agriculture as perceived by COEP (e).

The dominant perspective of the managers and experts of COEP of Fars province was closer to the deep ecology paradigm (total weight of 0.620) with a considerable difference from the other two points of view and was assigned the first rank (Fig. 4, Table 4). The top managers of the central office of environmental protection had environmental concerns at the higher level and this was consistent with the mission which has been defined for their organization. Eco-development (0.300) with moderate importance and finally frontier economics (0.080) with the least priority were placed at the next stages after deep ecology (Table 4). According to deep ecology as the dominant paradigmatic perspective of the managers and specialists of COEP, having respect for the environment is required due to its intrinsic value apart from the benefits derived by human and other organisms. Thus, everyone must practice environmental conservation. It is logical that these radical environmentalists prefer the basic assumptions of deep ecology perspective due to their personal red lines as well as the transcendental mission of the organization in conserving the natural resources and the whole nature. As it is seen in Fig. 4, deep ecology was ranked as first perspective with quit large space with the other two worldviews especially the frontier economics.

### 3.2 Analysis of the agricultural environmental management strategies

First, the findings from the opinions of five groups in terms of diverse types of environmental management strategies in agriculture of Iran have been presented. Next, the analysis of the comparison of these five groups was explained based on appropriate strategies of environmental management.

#### 3.2.1 Key policy makers of Agricultural Extension of the Ministry of Agriculture Jihad (AEMAJ)

Independent strategies (60.80) were chosen with the highest priority as the most appropriate executive strategies for environmental management of agriculture in Iran (Table 6). This was followed by cooperative strategies (49.42) and strategic maneuvering (37.20) in the next priorities (Table 6). The findings revealed that implementing of independent, separate and sporadic strategies was the main orientation of key agricultural extension policy makers at the ministry level suggesting the choice of a separate strategy for different challenges ahead. Based on the results, the strategies of “modern agricultural technologies to agricultural production increase” and “improvement of rural livelihood” were the most important strategies among all independent strategies in the opinion of agricultural extension policy makers of Iran (Table 5). On the other hand, an independent strategy of “privatization of resource property” and “educational programs of environmental management” were considered as the least priority by this group. Iranian policy makers of agricultural extension did not believe in the strategy of “decentralization and localization in agriculture” as an appropriate strategy to achieve agricultural environmental management and have given it the least priority among the other cooperative strategies (Table 5). Finally, national policy makers of this group did not favor strategic maneuvering as the third category of the strategies and have evaluated all items in this category as least important.

**Table 5:** Prioritization of environmental management strategies in accordance with the perspectives of the different groups.

Strategies	Items	Groups											
		AEMAJ		OAJ		ANRREC		ESOANR			COEP		
		Mean	Rank	Mean	Rank	Mean	Rank	Mean		Rank	Mean		Rank
Independent Strategies	Unit organization as a trustee for EM	4.32	7	4.17	3	4.03	6	4.07	6		3.90	9	
	Using modern agricultural technologies	4.90	1	4.14	4	4.07	5	4.16	4		4.10	6	
	Privatization of resources property	3.80	14	3.43	13	3.31	13	3.90	8		3.47	13	
	Improvement of rural livelihood	4.80	2	4.51	1	4.00	7	4.00	7		3.10	14	
	Educational programs of EM	3.90	13	4.12	5	4.10	3	4.10	5		4.04	7	
	Issues and leaflet about EM	4.27	9	3.91	8	3.69	10	3.50	11		3.83	11	
	No chemical input in agriculture	4.10	12	3.49	12	3.56	11	3.00	13		3.70	12	
	Biotechnology in agriculture	4.60	3	3.71	9	4.09	4	4.20	3		4.15	5	
	Land leveling to optimal water use	4.40	6	3.97	7	3.88	8	3.30	12		4.20	4	
	Organic fertilizers use in agriculture	4.20	10	4.20	2	4.13	2	3.70	10		4.30	3	
	Conservation of plant species by tissue culture	4.50	5	3.60	10	3.50	12	3.80	9		3.97	8	
	Collection of various seeds	4.30	8	3.57	11	3.72	9	4.30	2		3.87	10	
	Development of clean energy	4.51	4	4.11	6	4.28	1	4.40	1		4.53	1	
	Minimum external input in agriculture	4.14	11	3.31	14	3.09	14	2.80	14		4.47	2	
Cooperative Strategies	Cooperation of different organizations for EM	3.70	7	4.06	6	3.94	7	4.60	1		3.93	9	
	Information on optimal use of NR	4.10	3	4.17	4	4.13	4	4.26	4		4.17	4	
	Indigenous knowledge of local rural for EM	2.30	9	1.49	10	4.00	6	4.20	5		4.05	7	
	Development of entrepreneurship and employment in agricultural activities	3.60	8	4.23	2	3.81	9	3.80	10		3.97	8	
	Emphasize on biocapacity and NR thresholds	3.80	6	4.02	7	4.06	5	4.08	7		4.10	5	
	Comprehensive agricultural programs	4.00	5	4.14	3	3.88	8	3.90	8		4.07	6	
	Integrated management of pests, disease and weeds	4.08	4	4.08	5	4.16	3	4.10	6		4.30	2	
	Waste management and recycling	4.12	2	3.86	8	4.22	2	4.27	3		4.27	3	
	Management of renewable resources	4.20	1	4.49	1	4.41	1	4.30	2		4.40	1	
	Decentralization and localization in agriculture	1.50	10	2.23	9	3.79	10	3.85	9		3.77	10	
Strategic Maneuvering	Rural empowerment by NGOs	3.70	3	4.37	1	3.84	6	4.27	4		4.27	7	
	Green taxes	1.32	7	1.49	5	3.25	7	3.20	7		4.73	1	
	Green incentives	1.40	5	1.37	7	4.13	2	4.00	5		4.60	3	
	Social capital application in program planning	2.60	4	2.51	4	3.88	5	4.30	2		4.58	4	
	Strict environmental rules and regulations	1.30	6	1.40	6	4.00	3	3.80	6		4.63	2	
	Improvement of farmers' motivation and accountability for EM	3.65	2	4.26	3	3.91	4	4.20	3		4.33	6	
	Improvement of farmers' attitudes toward EM	3.80	1	4.30	2	4.34	1	4.60	1		4.40	5	

Scale: 1–5 EM = Environmental Management; NR = Natural Resources.

**Table 6:** Description of the five groups in terms of different environmental management strategies.

Groups	Strategies	Range	Minimum	Maximum	Mean
AEMAJ	Independent Strategies	10	56	66	60.80
	Cooperative Strategies	28	32	60	49.42
	Strategic Maneuvering	12	30	42	37.20
OAJ	Independent Strategies	30	38	68	54.29
	Cooperative Strategies	21	39	60	51.52
	Strategic Maneuvering	18	30	48	39.43
ANRREC	Independent Strategies	26	40	66	53.44
	Cooperative Strategies	29	41	70	56.57
	Strategic Maneuvering	36	34	70	54.69
ESOANR	Independent Strategies	26	37	63	53.20
	Cooperative Strategies	28	42	70	57.96
	Strategic Maneuvering	30	40	70	56.80
COEP	Independent Strategies	31	39	70	55.80
	Cooperative Strategies	34	36	70	57.49
	Strategic Maneuvering	18	52	70	63.13

The same scores have been assigned to all three categories providing the same condition for comparisons (scale: 14–70).

#### 3.2.2 Top managers and authorities of Organizations of Agricultural Jihad (OAJ) at provincial level

Independent strategies (54.29) had the highest priority in the viewpoints of the top managers and executives of OAJ (Table 6). “Improvement of rural livelihood” and “Organic fertilizers use in agriculture” were considered to be the most important among all independent strategies in the opinion of this group (Table 5). The top managers of Organizations of Agriculture Jihad following their superiors at the ministry level, prioritized the cooperative strategies (51.52) and strategic maneuvering (39.43) at the next ranks (Table 6). “Management of renewable resources” and “development of entrepreneurship and employment in agricultural activities” were the most important in the category of cooperative strategies. The top managers of OAJ did not believe in “indigenous knowledge of local rural for environmental management” and “decentralization and localization in agriculture” among cooperative strategies and gave them the least priorities in this category. Lastly, the strategy of “rural empowerment by NGOs” was considered as the highest priority in the strategic maneuvering category, but the remaining items received low scores (Table 5).

#### 3.2.3 Top managers and researchers of Agricultural and Natural Resources Research and Education Centers (ANRREC) of provinces

The experts of this group have given the highest priority to cooperative strategies (56.57). This was followed by strategic maneuvering (54.69) and independent strategies (53.44) with little difference between the two (Table 6). Indeed, the managers and researchers of ANRREC in the studied provinces considered all three types of strategies as appropriate solutions to comprehensive environmental management of agriculture but they had strong orientation to cooperative strategies as opposed to the other two categories. “Development of clean energy application” and “organic fertilizers use in agriculture” were the most important strategies among the all independent strategies according to the top managers and researchers of ANRREC in the four provinces of the study (Table 5). The cooperative strategies of “management of renewable resources”, “waste management and recycling” and “integrated management of pest, disease and weeds” received the highest scores by this group of experts. Finally, the strategies of “improvement of farmers' attitudes toward environmental management” and “green incentives to active agricultural firms” were considered the highest priorities in the strategic maneuvering category (Table 5).

#### 3.2.4 Council of Engineering System Organization of Agriculture and Natural Resources (ESOANR) of Fars province

Similar to the previous group, the experts of the council of ESOANR ranked cooperative strategies (57.96), strategic maneuvering (56.80) and independent strategies (53.20) as appropriate strategies for sustainable environmental management in agriculture of Iran, respectively (Table 6). “Development of clean energy application,” “Collection of various seeds” and “biotechnology in agriculture” were the most important independent strategies (Table 5). “Cooperation of different organizations for environmental management” and “management of renewable resources” were selected as the most effective cooperative strategies in order to achieve sustainable agricultural development. Finally, “improvement of farmers' attitudes toward environmental management” and “social capital application in program planning” were assigned as the highest priorities in the strategic maneuvering category by the key authorities of agricultural private sector of Iran (Table 5).

#### 3.2.5 Top managers and the think tank members of Central Office of Environmental Protection (COEP) of Fars province

Strategic maneuvering (63.13) had the highest priority according to the viewpoint of heads and experts of think tank of COEP of Fars province (Table 6). “Green taxes,” “strict environmental rules and regulations” and “green incentives to active agricultural firms” were chosen as the most effective strategies among the other items of the strategic maneuvering category by the experts of this group (Table 5). Cooperative strategies (57.49) and independent strategies (55.80) were placed after strategic maneuvering (Table 6). “Management of renewable resources” and “integrated management of pests, disease and weeds” as cooperative strategies and “development of clean energy application” and “minimum use of external input in agriculture” as independent strategies were the most important by the heads and think tank experts of COEP of Iran (Table 5).

The results demonstrate different rankings by the five groups of the study in terms of different types of environmental management strategies, but there were some similarities in this regard. Even though the three groups of ANRREC, ESOANR and COEP accorded close scores to three categories of strategies and considered all of them as the effective and appropriate solutions for environmental management, their tendency and orientation favored strategic maneuvering and cooperative strategies. These three groups believed that the application of independent and sporadic strategies no longer works and it is necessary to move toward integration of different types of strategies as well as stakeholders' empowerment in order to achieve sustainable environmental management. On the contrary, independent strategies were considered as the highest priority by the other two groups of AEMAJ and OAJ in the study.

#### 3.2.6 Comparing different groups in terms of environmental management strategies

To provide more accurate comparisons among the opinions of different groups in terms of executive strategies of agricultural environmental management of Iran, an analysis of variance (ANOVA) test was performed. Three ANOVAs were assessed due to different types of strategies. Based on the results for independent strategies, there was a significant difference between the score mean of this type of strategy in the perspective of agricultural extension key policy makers of the ministry and the other groups (sig. = 0.041). The score mean of independent strategies based on key policy makers of the ministry (60.80) was significantly higher (Table 7).

**Table 7:** ANOVA results for comparison of strategies among groups.

Groups	Strategies					
	Independent Strategies		Cooperative Strategies		Strategic Maneuvering	
	Mean	SD	Mean	SD	Mean	SD
AEMAJ	60.80^a^	3.01	49.42^a^	11.06	37.20^a^	4.73
OAJ	54.29^b^	6.16	51.52^a^	5.73	39.43^a^	3.61
ANRREC	53.44^b^	6.91	56.57^b^	8.51	54.69^b^	10.48
ESOANR	53.20^b^	8.41	57.96^b^	9.79	56.80^b^	10.29
COEP	55.80^b^	7.76	57.49^b^	7.62	63.13^c^	5.55
F	2.582		4.327		55.917	
Sig.	0.041		0.003		0.0001	

The means denoted with similar letters were not significantly different at the 0.05 level in the LSD test.

The results of an ANOVA regarding cooperative strategies revealed that there was a significant difference between the score mean of the opinions of the two groups of AEMAJ and OAJ and the other three groups (sig. = 0.003). The score mean of cooperative strategies in the groups of ANRREC (56.57), ESOANR (57.96) and COEP (57.49) were greater than the other two groups (AEMAJ, OAJ) (Table 7). Based on the ANOVA test regarding strategic maneuvering, there was also a significant difference between the score mean of AEMAJ and OAJ and the other three groups in terms of strategic maneuvering scores (sig. = 0.0001). The score means of strategic maneuvering were 37.20 and 39.43, respectively, which were less than the other groups (Table 7). There was a significant difference for the score of strategic maneuvering of the experts of two groups of ANRREC and ESOANR and the other groups. The score means of these two groups at the moderate level equal 54.69 and 56.80, respectively. Finally, there was a significant difference in the score mean of strategic maneuvering between the viewpoint of managers and think tank members of COEP and the other four groups in the study; the score mean of this type of strategy with the highest amount equals 63.13. The results of ANOVA tests including the means, standard deviation, F and significance levels are presented in Table 7.

## 4 Conclusion

The paradigms constitute the intellectual foundation that includes the values, beliefs and norms of the individual, organization or society. Since strategies and decisions are also rooted in the intellectual foundation, one can perceive the strategies and behaviors of the individual or organization by reviewing their intellectual paradigm. The strategies which are selected and implemented by policy makers, executives and managers are also extracted from their paradigmatic viewpoints. Frontier economics, eco-development and deep ecology are considered as the three fundamental paradigmatic perspectives relevant to environmental management debates. Frontier economics proponents, with emphasis on economic components, consider economic growth and high agricultural production as the main solution to sustainable agricultural development. In contrast, radical environmentalists believe in deep ecology and consider it as the highest priority for environmental protection under any circumstances. Finally, there is a moderate and intermediate perspective called eco-development, which takes into consideration economic, social and environmental aspects in order to achieve comprehensive environmental management. There are three different types of strategies in terms of environmental management which are matched with different paradigmatic perspectives. Regarding complexity, these strategies are independent strategies, cooperative strategies and strategic maneuvering, respectively. The environmental management strategies selected by each principal group of the agricultural sector of Iran are consistent with their paradigmatic viewpoints.

The key national policy makers and executives of agricultural extension of Iran have an economic paradigmatic perspective toward environmental management, thus assigning priority to economic factors such as natural resources use to meet the needs of Iranians more than the other socio-environmental elements. Removing economic barriers, agricultural production growth and more utilization of natural resources are the most effective factors to sustainable agricultural development based on the opinions of the agricultural extension policy makers and executive managers. Consistent with this intellectual foundation, selected strategies would be mostly independent, separate and sporadic. In other words, the orientation of the principal agricultural extension policy makers of Iran supports implementation of reactive strategies after facing the crisis. Temporary and positional reduction of environmental damage in the agricultural sector is the main objective of this kind of strategy.

The managers and policy makers of the private sector, as well as researchers of agriculture of Iran, have a moderate paradigmatic viewpoint, so they consider socio-environmental aspects in addition to economic factors, and even give highest priority to environmental aspects most of the time. They also have a greater tendency to support integrated and cooperative strategies in addition to reactive independent strategies in special situations. Agricultural researchers and the policy makers of the private sector prefer proactive strategies over reactive ones. They believe in foresight and implementation of appropriate strategies before facing environmental degradation as a means of crisis prevention. Using proactive strategies in agriculture requires systematic land use planning studies in order to identify the capacities of natural resources of each region for comprehensive land and water management. Thus it could be possible to determine and cope with environmental challenges and barriers before a crisis occurs.

Environmental policy makers, superior managers and the specialists of Fars province have a totally different perspective in comparison with the other agricultural policy maker groups of the study. This group has perceived intensive environmental crisis as well as strong environmental concerns more than others. They absolutely believe in prioritizing the environmental components, so they prefer far more complex strategies for comprehensive environmental management in order to achieve sustainable agricultural development. The policy makers and experts of the central office of environment protection argue that it is necessary to rethink, modify and redesign common strategies in order to resolve environmental challenges and crises of Iran. Using new strategies in the agriculture sector leads to maximum consistency between the environmental programs and challenges, as well as adjusting to the specific condition of each region. On the other hand, it is possible to change and modify the strategies in accordance with their flexibility. Empowerment of rural farmers enables them to confront independently future problems in terms of environmental challenges to agriculture.

The paradigmatic perspectives of the agricultural extension key policy makers of Iran suggest that the viewpoint of the main policy makers and authorities as the superior executives of agricultural extension of the country is mainly supports agricultural production growth due to the modernization and diffusion of agricultural innovations theory. Environmental crisis would be worsened following the continuity of this theory. In contrast, the managers and specialists of the other organizations related to agriculture have more moderate perspectives related to the environmental circumstances, but they do not have much executive force to make necessary changes in the country. The paradoxes among the paradigmatic viewpoints of different policy maker groups in agriculture sector of Iran is another challenge which reduces their effective interactions. Weak communications among these groups of policy makers is probably rooted in the strong differences in their perspectives and intellectual paradigms. Selecting and implementing effective strategies for better environmental management of agriculture has not happened since there is no change in the paradigmatic perspectives of agricultural policy makers and executives of Iran. A paradigm shift is required from frontier economics to the more environmental paradigm of eco-development in the age of environment called ecological enlightenment due to the widespread environmental crisis. Thus, a fundamental change is needed in the dominant theory of innovation diffusion focusing on economic factors to the green theories emphasizing all aspects of socio-economic and environmental components. Emphasizing on economic growth and agricultural production increase through transfer of technical knowledge no longer work. It is also required to notice biocapacity of resources, poverty alleviation, environmental sustainability, food safety and multi-functional agriculture comprehensively to a sustainable environmental management.

## Declarations

### Author contribution statement

Mahsa Fatemi: Conceived and designed the experiments; Performed the experiments; Analyzed and interpreted the data; Contributed reagents, materials, analysis tools or data; Wrote the paper.

Kurosh Rezaei-Moghaddam: Conceived and designed the experiments; Contributed reagents, materials, analysis tools or data; Wrote the paper.

### Funding statement

This research did not receive any specific grant from funding agencies in the public, commercial, or not-for-profit sectors.

### Competing interest statement

The authors declare no conflict of interest.

### Additional information

No additional information is available for this paper.
